# The correlation between chronic sinusitis and chronic adenoiditis in children

**DOI:** 10.1186/2045-7022-3-S2-P17

**Published:** 2013-07-16

**Authors:** Felicia Manole

**Affiliations:** 1 Faculty Of Medicine Oradea, Oradea, Romania; 2 Faculty Of Medicine Oradea, ENT, Oradea, Romania

## Background

The purpose of the study is to evaluate the correlation between chronically sinusitis and chronically adenoiditis in children.

## Methods

The study was carried out on two groups of children aged between 6 and 18, diagnosed and treated in the Pediatric Hospital of Oradea in the period 2010-2012. In the first phase of the study, diagnosis was established on clinical and radiological criteria. The first group of 112 patients was submitted to adenoidectomy. The second group of 40 patients was given medicine instead of being submitted to surgical procedures. In the second phase, six months later, patients were re-evaluated clinically and with endoscopy.

## Results

70% of the patients were aged between 10-14 in both groups. There are no statistic differences between the two groups. In both groups the study showed the predominance of ethmoido-maxillary sinusitis. Nasal obstruction prevailed in the case of the first group (surgically treated-adenoidectomy). Rhinorrhea was almost equally distributed in the first phase at patients in both groups; on second evaluation, we noticed its absence in 60% of patients in the first group. Statistically, the difference between the two groups, in the case of second evaluation, is strongly significant. On second evaluation, in the case of the first group, the study showed the reduction of sinus infection in 56% of patients as compared to the second group, where recovery was present in only 24% of patients.

## Conclusions

As a result of adenoidectomy, the reduction of the inflammation in the case of sinus infection, can be a proof of the role of the nasopharynx. Chronic hypertrophic adenoiditis can be both a mechanical obstacle and a cause of infection of the paranasal sinuses in children.

